# The preliminary study for three-dimensional alveolar bone morphologic characteristics for alveolar bone restoration

**DOI:** 10.1186/s40902-019-0216-2

**Published:** 2019-09-05

**Authors:** Hyun-Jae Cho, Jae-Yun Jeon, Sung-Jin Ahn, Sung-Won Lee, Joo-Ryun Chung, Chang-Joo Park, Kyung-Gyun Hwang

**Affiliations:** 1 Division of Oral & Maxillofacial Surgery, Department of Dentistry, College of Medicine, Hanyang University, 222 Wangsimni-ro, Seongdong-gu, Seoul, 04763 South Korea; 2 Division of Oral & Maxillofacial Surgery, Department of Dentistry, Hanyang University Medical Center, Seoul, South Korea

**Keywords:** Alveolar bone morphology, CT, Guided bone regeneration, Dental implant, Width and length of teeth

## Abstract

**Background:**

The concept of the ideal morphology for the alveolar bone form is an important element to reconstruct or restore the in maximizing esthetic profile and functional alveolar bone restoration. The purpose of this preliminary study is to evaluate the normal alveolar bone structure to provide the standard reference and guide template for use in diagnosing for implant placement, determining the correct amount of bone augmentation in actual clinical practice and producing prostheses based on three-dimensional imaging assessment of alveolar bone.

**Methods:**

This study was included 11 men and 11 women (average age, 22.6 and 24.5 years, respectively) selected from among 127 patients. The horizontal widths of alveolar bone of maxilla and mandible were measured at the crestal, mid-root, and root apex level on MDCT (multi-detector computed tomography) images reconstructed by medical imaging software. In addition, tooth dimensions of the central incisors, canines, second premolars, and first molars of maxilla and mandible, including the horizontal width of the interdental alveolar bone crest, were also measured and statistically analyzed.

**Results:**

The horizontal alveolar bone width of the palatal side of maxilla showed a distinct increment from the alveolar bone crest to the apical region in both anterior and posterior areas. The average widths of the maxillary alveolar ridge were as follows: central incisor, 7.43 mm; canine, 8.91 mm; second premolar, 9.57 mm; and first molar, 12.38 mm. The average widths of the mandibular alveolar ridge were as follows: central incisor, 6.21 mm; canine, 8.55 mm; second premolar, 8.45 mm; and first molar, 10.02 mm. In the buccal side, the alveolar bone width was not increased from the crest to the apical region. The horizontal alveolar bone width of an apical and mandibular border region was thinner than at the mid-root level.

**Conclusions:**

The results of the preliminary study are useful as a clinical guideline when determining dental implant diameter and position. And also, these measurements can also be useful during the production of prefabricated membranes and customized alveolar bone scaffolds.

## Background

Guided bone regeneration (GBR) is a well-defined method of alveolar bone augmentation [1]. Although, alveolar bone augmentation should be restored the pre-existing alveolar bone contour for implant placement, dentist has performed ridge augmentation with rough estimate about alveolar bone morphology based on subjective assessment or the adjacent tooth and perioontal structure. These may lead to an unsatisfactory esthetic result in the anterior region and insufficient functional restoration in the posterior region [2]. However, objective standards for the amount of bone graft are unclear, and in cases of extensive alveolar bone reconstruction, no adjacent alveolar bone is available for reference. The concept of the ideal morphology for the alveolar bone form is an important element to reconstruct or restore the in maximizing esthetic profile and functional alveolar bone restoration. This concept is also an essential factor in oral and maxillofacial tissue engineering field. The development of 3D printing technology is accelerating the customized alveolar bone scaffold manufacture with various bio-material for alveolar bone restoration [3].

The need for the ideal morphology of alveolar bone has been increasing on dental implant and maxillofacial field. There have been studies examining the resorption of alveolar bone and the normal form of alveolar bone [4]. However, previous studies were focused on the direction and pattern of alveolar bone resorption with two-dimensional image based on cadaveric maxillae and mandibules. The development of digital dentistry can make a three-dimensional reconstruction of conventional computed tomography (CT). The CT data (DICOM) processing software produces three-dimensional images that make it possible to establish the best alveolar position for implant placement and to assess the morphology of the alveolar bone [5]. Creating the three-dimensional image of the desired shape of alveolar bone using CT image and forecasting bone-graft requirements would improve the success rates of implant placement and aid in esthetic and functional recovery.

**Fig. 1:**
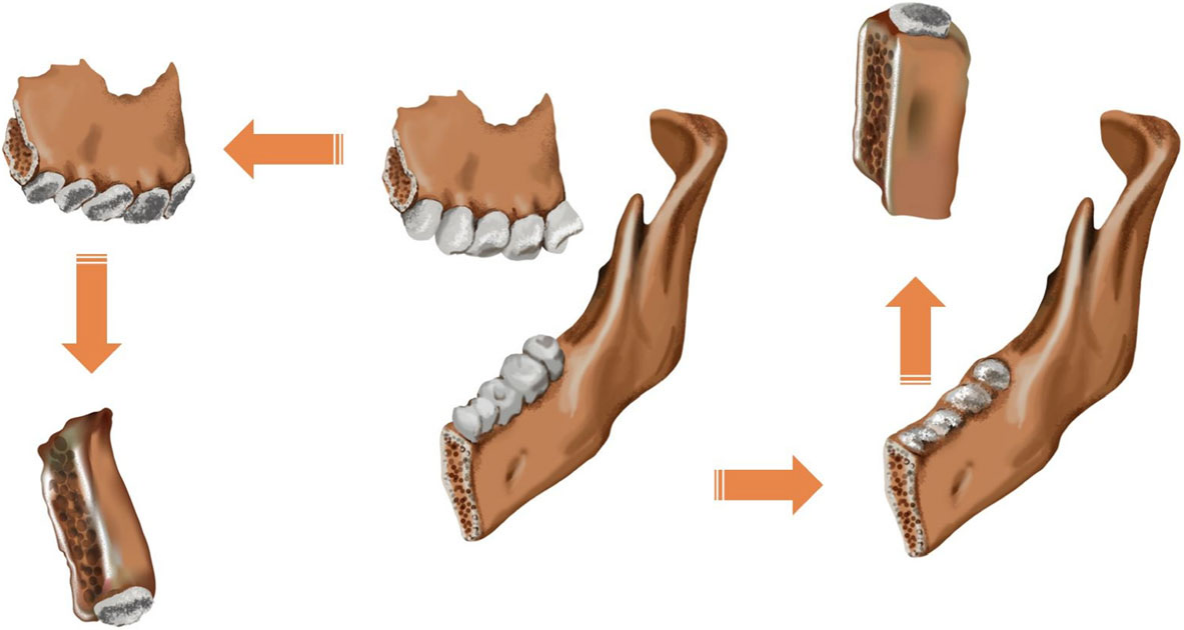
Evaluation of the 3D alveolar bone morphology. The concept of this preliminary study is to evaluate the ideal three-dimensional alveolar bone morphology by dividing the single tooth block on 3D reconstruction maxilla and mandible

The purpose of this preliminary study is to evaluate the normal alveolar bone morphology to provide the standard reference and guide template for use in diagnosing for implant placement, determining the correct amount of bone augmentation in actual clinical practice and producing prostheses based on three-dimensional imaging assessment of alveolar bone (Fig. 1).

## Materials and methods

### Patients

The study included 11 men and 11 women, average age 22.6 and 24.5 years, respectively (Table 1), who were selected from among 127 patients who underwent multi-detector computed tomography (MDCT, Sensation 16, Siemens Medical Solutions, Forchheim, Germany) at the Department of Dentistry of Hanyang University Medical Center. Exclusion criteria were facial asymmetry, periodontal diseases, alveolar bone pathology, and history of prior orthodontic treatment.

**Table 1:** Patient characteristics

	Male	Female
Subject (*n*)	11	11
Age (year)	22.6 ± 2.29	24.5 ± 3.41
Range (year)	20–26	20–29

*n* number

### Ethical approval

The institutional review board of the College of Medicine, Hanyang University approved the study (HY-14-023-2). Informed consents have waived in this study, since there was no conflict of interest and additional payment or visiting for patients.

### Data collection

Data were extracted retrospectively from MDCT images stored as Digital Imaging and Communications in Medicine (DICOM) files. The images were sliced at 1-mm intervals. Medical imaging software (OnDemand 3D^®^, Cybermed, Seoul, Korea) was used to measure and evaluate the alveolar bones of unilateral maxillary and mandibular central incisors, canine teeth, second premolars, and first molars. Informed consent was waived because of the retrospective nature of the study.

### Measurement of alveolar bone morphology

#### Horizontal width of alveolar bone

**Fig. 2:**
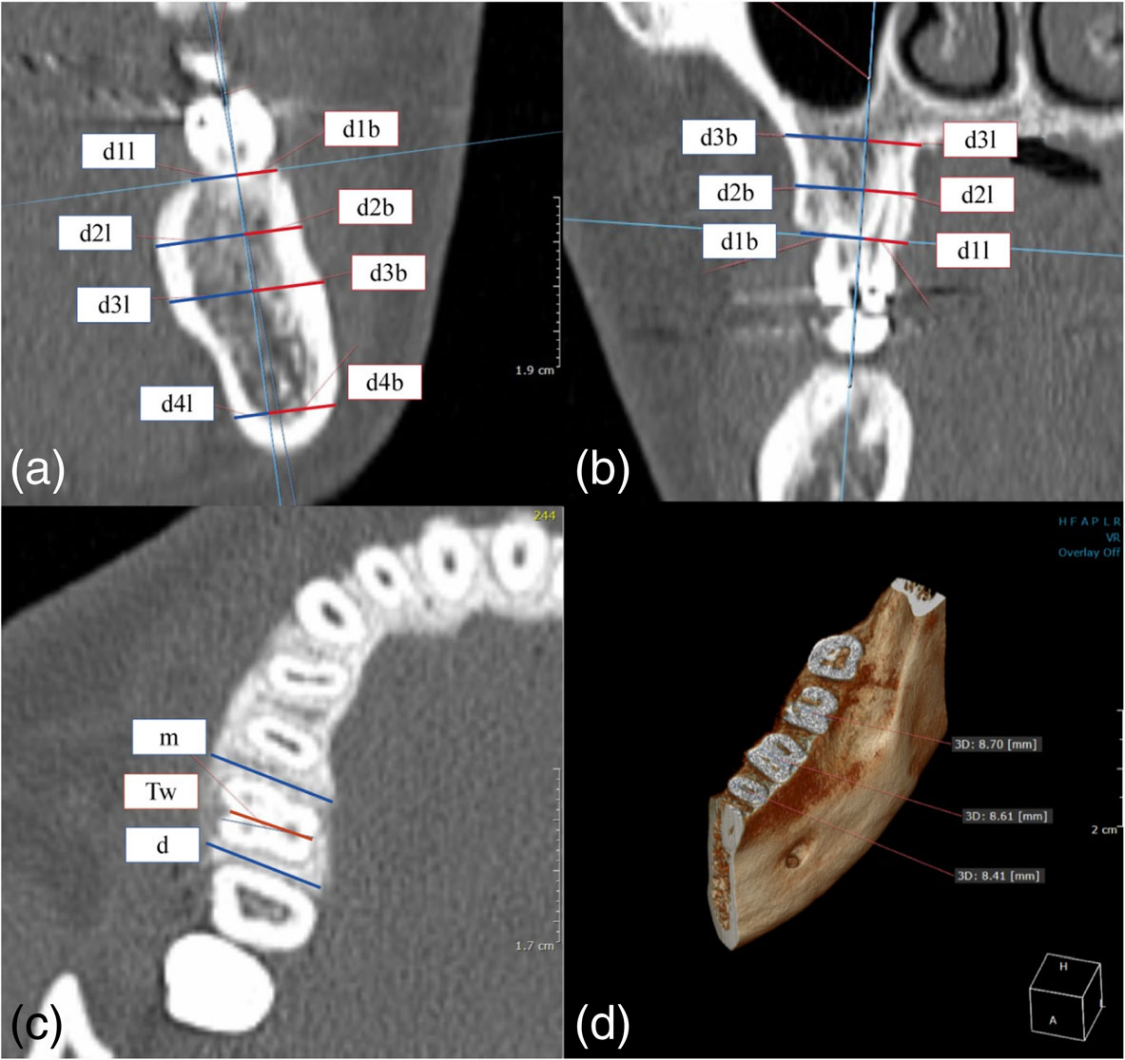
Measurement of horizontal width of alveolar bone. **a** Working on a coronal view of the mandibular first molars. **b** Working on a coronal view of the maxillary first molars. **c** Measurement of the horizontal width of the interdental alveolar bone crest (m, d) and the teeth (Tw). **d** Working on volumetric view on mandibular first molars. d1 = bone width at the crestal level; d2 = bone width at the mid-root level; d3 = bone width at root the apex level; d4 = bone width at the mandibular border level. b, l = buccal and lingual sides; m, d = mesial and distal sides; Tw = width of tooth at the alveolar bone crest level

The DICOM files of each patient were analyzed using the medical imaging software. After setting horizontal and vertical coordinate systems to the axis of the tooth for measurement in three-dimensional space, the width of the alveolar bone [*d1*] on the plane parallel to the bucco-lingual distance at the alveolar crest on the sagittal section and the width of the alveolar bone 5 mm from the apex of the root [*d3*] and parallel to the bucco-lingual alveolar bone crest were measured. The width of the alveolar bone midway between the alveolar bone crest and the apex area [*d2*] was also measured, and the width of the mandible at 5 mm from the inferior mandibular border parallel to the crest was measured [*d4*]. The widths of each buccal bone surface were identified as *d1b*, *d2b*, *d3b*, and *d4b*, and the widths of each lingual bone surface were identified as *d1l*, *d2l*, *d3l*, and *d4l*. The buccal and lingual sides were divided from the point of intersection of the lines connecting the axis of the tooth and the alveolar crest (Fig. 2)

#### Thickness of interdental alveolar bone

The width of the horizontal alveolar bone at the mesial side of the alveolar crest region inferior to the tooth contact point was identified as [*m*], and the width of the alveolar bone on the distal side was defined as [*d*]. After marking a line parallel to the bucco-lingual direction of the tooth at the level of the tooth contact point, the thickness of the alveolar bone was measured.

#### Measurement of width and length of teeth

The width of the tooth at the center of the axial bisector of the tooth in the sagittal plane at alveolar bone crest level was measured, and this value was defined as [*Tw*] (Fig. 2c). The length of tooth, as the distance from the tooth section or buccal cusp tip to the apex, was measured and defined as [*Tl*].

### Statistical analysis

Statistical analyses were performed using SPSS version 20.0 (IBM, Chicago, IL, USA). The mean values of alveolar bone and tooth measurements were calculated. Kruskal-Wallis test was then applied to detect significant differences by sex and, analysis of variance (ANOVA) and Kruskal-Wallis test were performed to verify statistical differences along the measurement point. *P* < 0.05 indicates statistical significance.

## Results

### Horizontal width of alveolar bone

**Fig. 3:**
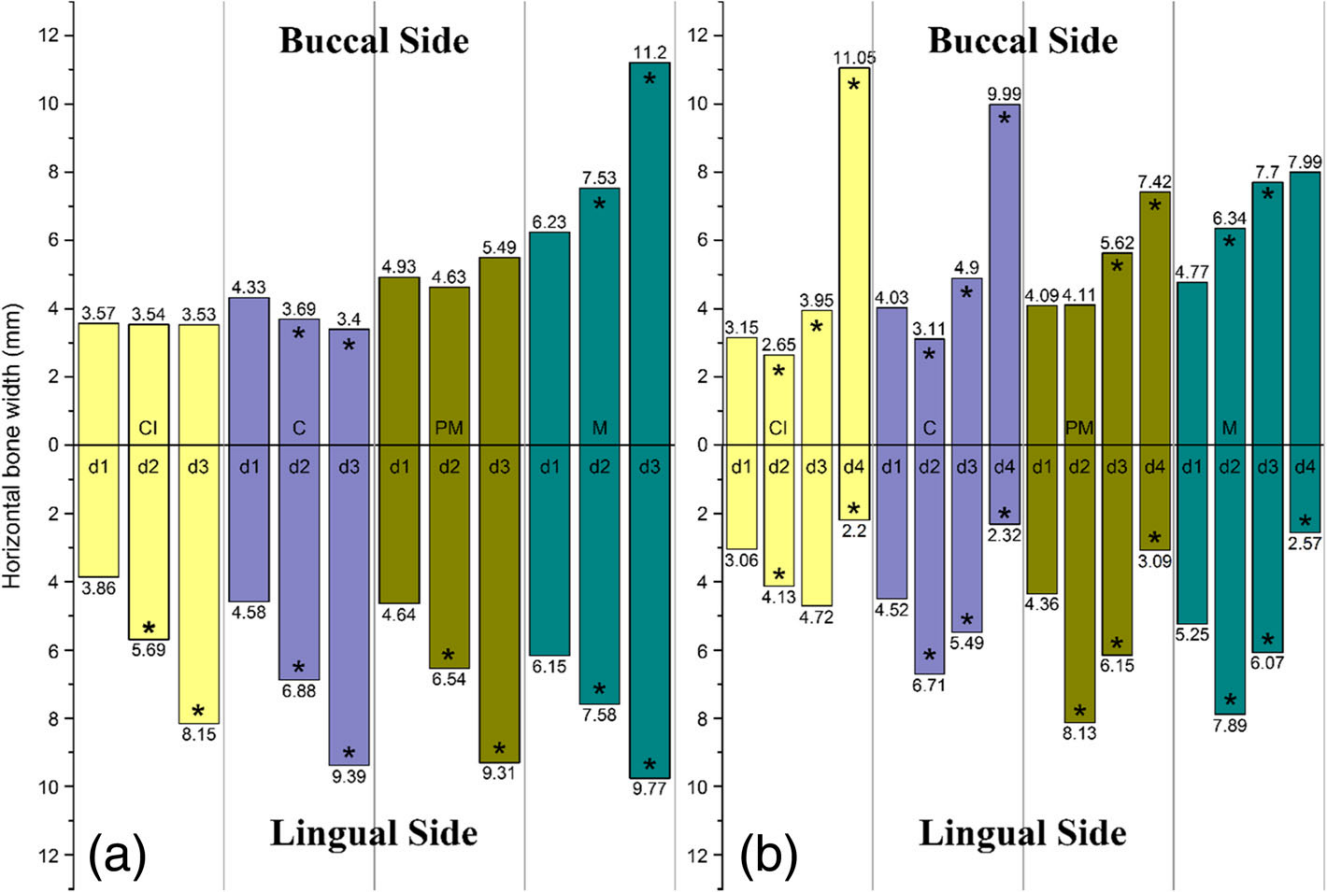
Comparison of horizontal alveolar widths between **a** maxillary and **b** mandibular alveolar bone. CI, central incisors; C, canines; PM, premolars; M, molars; d1 = bone width at the crestal level; d2 = bone width at the mid-root level; d3 = bone width at root the apex level; d4 = bone width at the mandibular border level (*P < 0.05)

The mean horizontal width values for alveolar bone and size of teeth are given in Table 2. The horizontal alveolar bone width of the palatal side of maxilla showed a distinct increment from the alveolar bone crest to the apical region in both anterior and posterior areas. In the buccal side, the alveolar bone width was not increased from the crest to the apical region. There was a lingual concavity at the mandibular canine, second premolar, and first molar sites (Fig. 3b). The horizontal alveolar bone width of an apical and mandibular border region was thinner than at the mid-root level.

**Table 2:** Horizontal width of alveolar bones (d1, d2, d3, d4), (m, d), and tooth dimensions (Tw, Tl)

		d1b	d1l	d2b	d2l	d3b	d3l	d4b	d4l	m	d	Tw	Tl
Mx.	CI	3.57	3.86	3.54*	5.69	3.53	8.15	N/A		5.73	5.90	6.30	22.57
		(± 0.448)	(± 0.361)	(± 0.485)	(± 0.935)	(± 1.163)	(± 1.644)			(± 0.836)	(± 0.471)	(± 0.430)	(± 1.626)
	C	4.33	4.58*	3.69*	6.88	3.40	9.39			6.12*	7.45*	7.68	24.31
		(± 0.715)	(± 0.451)	(± 0.931)	(± 4.027)	(± 1.498)	(± 2.758)			(± 0.841)	(± 0.722)	(± 1.187)	(± 3.312)
	PM2	4.93	4.64	4.63	6.54	5.49	9.31			8.03*	9.42*	8.22	19.00
		(± 0.492)	(± 0.455)	(± 0.984)	(± 1.039)	(± 2.437)	(± 1.598)			(± 0.728)	(± 0.759)	(± 0.574)	(± 1.830)
	M1	6.23	6.15	7.53	7.58	11.20	9.77			9.50*	12.21	10.00	19.06
		(± 0.559)	(± 0.522)	(± 0.897)	(± 0.809)	(± 2.470)	(± 1.519)			(± 0.709)	(± 1.166)	(± 0.621)	(± 1.504)
Mn.	CI	3.15	3.06	2.65	4.13	3.95	4.72*	11.05	2.20	4.66*	5.43*	5.65	20.08
		(± 0.430)	(± 0.215)	(± 0.375)	(± 0.703)	(± 1.228)	(± 1.140)	(± 2.107)	(± 1.647)	(± 0.676)	(± 0.542)	(± 0.396)	(± 1.334)
	C	4.03*	4.52*	3.11*	6.71*	4.90	5.49*	9.99*	2.32	6.45	6.60	7.60*	24.83
		(± 0.535)	(± 0.628)	(± 0.499)	(± 1.372)	(± 1.364)	(± 1.268)	(± 1.901)	(± 0.769)	(± 0.892)	(± 0.847)	(± 0.879)	(± 1.657)
	PM2	4.09	4.36	4.11	8.13*	5.62	6.15*	7.42	3.09	6.84	8.10	7.15	21.20
		(± 0.432)	(± 0.667)	(± 0.752)	(± 1.547)	(± 1.304)	(± 2.183)	(± 1.852)	(± 1.544)	(± 1.115)	(± 0.920)	(± 0.556)	(± 1.805)
	M1	4.77	5.25	6.34	7.89*	7.70	6.07	7.99	2.57	8.25	9.12	7.91	19.43
		(± 0.420)	(± 0.550)	(± 0.942)	(± 1.172)	(± 1.230)	(± 1.702)	(± 1.554)	(± 1.054)	(± 1.045)	(± 0.918)	(± 0.674)	(± 1.865)

*CI* central incisors, *C* canines, *PM2* second premolars, *M1* first molars, *d1* bone width at the crestal level, *d2* bone width at the mid-root level, *d3* bone width at root the apex level, *d4* bone width at the mandibular border level, *b, l* buccal and lingual surfaces, *Tw* width of tooth at the cementoenamel junction

*Statistically significant differences are marked between the sexes (*P* < 0.05, Mann-Whitney test)

### Thickness of interdental alveolar bone

1. Central incisors of maxilla and mandible

There was no significant difference in horizontal alveolar bone widths between the crest, mid-root, and apical areas on the buccal side, whereas there was a significant difference on the lingual side. The buccal bone width at crest level of the maxillary anterior region was 0.42 mm after subtracting a root portion of the central incisor. The buccal bone widths remain thin constantly, as it progresses from the crest to the apical region, while the palatal bone width increases (Fig. 3a). There was a concavity at the mid-level of the root at the buccal side on the mandible (Fig. 3b). The buccal alveolar bone width at the mid-level was thinner than the crest and apical region significantly (*P* < 0.05). The width of the buccal alveolar bone was greater than that of the lingual bone at the border of the mandibular body because of the mandibular tubercle.

## Canines of maxilla and mandible

The thickness of the buccal bone of maxillary canines surface decreases significantly (*P* = 0.006) in the direction of the apical region but the palatal bone showed a distinct increment. As with the mandibular central incisors, the alveolar bone of the mandibular canine was thinner at the middle level of the roots than other regions significantly (*P* < 0.05). The buccal alveolar bone of the mandibular canine was concave and the general width increased toward the apex of the root.

## Second premolars of maxilla and mandible

In the maxillae, the thickness of lingual bone increased, whereas buccal bone was not distinctly changed. There was no statistically significant concavity at the mid-level of the roots (*P* > 0.05). The lingual alveolar bone was thicker at mid-root than at the crest and became thinner toward the mandibular border. However, the buccal alveolar bone was thicker at the mandibular border, and the measurements showed a concavity on the lingual side of the mandible.

## First molars of maxilla and mandible

The average length of the maxillary and mandibular first molar tooth was 19.06 and 19.43 mm, and the average width was 10.00 mm and 8.07 mm, respectively. The alveolar width at the alveolar crest of the maxillary and mandibular first molars region was 12.38 mm and 10.02 mm, respectively. Unlike the other forms of maxillary alveolar bone, the buccal alveolar bone of the first molar showed a distinct incremental change in width in the direction of the apical area (*P* < 0.05). The buccal alveolar bone of the mandibular first molars showed an increment from the crest to the apical region. The measurements showed a concavity on the lingual side like other lingual aspects of the mandible.

### Correlation of alveolar bone width and tooth size with sex

Non-parametric statistical analysis (Kruskal-Wallis test) was used to check for statistical significance of measurement differences between the sexes. In general, there were no significant differences of measurements of the maxillary teeth, but gender was significantly associated with differences in the width of the interproximal alveolar bone crest of the maxillary canines, second premolars, and mandibular central incisors. There was no significant difference between the sexes in the sizes of the maxillary teeth. In the case of mandibular canine, there was a significant difference on the alveolar bone crest, mid-root area, and apical area. The size of the tooth was not significantly different between sex except mandibular canine.

## Discussion

This study investigated the average horizontal width of normal buccal, palatal, and inter-dental alveolar bone by examining the form of the alveolar bones in male and female in the third decade of life (average age 22.6 years, men, and 24.5 years, women) who had normal periodontal status. It is important to determine ideal alveolar bone morphology for bone grafting procedures to establish a criterion for the amount of augmentation and to estimate the shape of the prosthesis and the amount of alveolar bone that will function when the bite force is applied. Several studies have been analyzed the width of the alveolar bone. However, the previous studies have measured the alveolar width simply on the basis of the tooth axis and have not provided correlations with the overall shape or size of the alveolar bone and the shape of the interdental alveolar bone [6]. The present study assessed the shape and dimensions of the alveolar bone, the size of the teeth, and the correlation of these parameters. In addition, this study has approximated an ideal normal shape of the alveolar bones by examining periodontally healthy adults in their 20s to exclude age-relating changes in the alveolar bones.

The average thickness of the buccal alveolar bone of the maxillary anterior teeth was 0.42 mm in this study. This is similar to the result of Januario et al. demonstrating that the facial bone in the anterior region was < 1 mm thick and that > 50% of it was < 0.5-mm thick [7]. Kim et al. studied the relationship between the maxillary incisor roots and surrounding alveolar structures on micro-CT. They indicated that maxillary incisors and canine root were placed at the labial one fifths areas [8]. Huynh-Ba et al. measured the bone wall dimensions of the post-extraction socket of maxillary anterior teeth and premolars [9]. They found that 87% of the buccal bony walls had a width ≤ 1mm and 64.1% had a width ≤ 0.5 mm. Vera et al. also measured the thickness of the buccal bone in the upper anterior jaw and at the premolar tooth in 43 patients and found that the thickness was 0.83 mm in the upper jaw anterior region and 1.13 mm at the premolar region at the level of the alveolar crest, and 0.70 mm and 0.70 mm, respectively, in the anterior area and at the mid-root level of the premolar [6]. This supports the assertion that because the buccal alveolar bone is very thin in the anterior upper jaw, careful attention to buccal bone preservation is critical to ensure the favorable esthetic and functional outcome of dental implants. Implant placement is preferably focused on the palatal position after the preliminary tooth extraction is carried out.

Braut et al. measured the thickness of the lingual alveolar bones of mandibular premolars and molars and of the horizontal bone in the alveolar bone crest area [10]. They found an averaged bone width of 7.63 mm at the horizontal bone of the alveolar bone crest of the second mandibular premolar, with the first molar having an average width of 10.34 mm. The average alveolar bone width at crest level was 8.45 mm for the second mandibular premolar and 10.02 mm for the first mandibular molar in this study.

The average width of the first molar in the present study was 10.00 mm in the maxilla and 7.91 mm in the mandible, while the horizontal width of the alveolar bone at the alveolar bone crest region was 12.38 mm in the maxilla and 10.02 mm in the mandible. This supports the finding of the systematic review by Van der Weijden et al. that is necessary to determine the site of lingual positioning of an implant with regard to some degree of horizontal bone resorption, because the extraction socket has a reported 3.87 mm of horizontal bone resorption and approximately 1.67 to 2.03 mm of vertical bone resorption [11].

Park et al. have demonstrated that the shape of the ridge has more influence on the outcome of bone grafting than the width [12]. The results of this study suggested that bone grafting might have a poorer outcome in the anterior region of the mandible because the angle of the cross-sectional ridge is narrow, while outcomes may be better in areas with a wide angle at the cross-sectional ridge, as in the mandibular molar area. There have been many studies on the extent of alveolar bone resorption, but few have focused on normal alveolar bone using CBCT to determine the horizontal width of the alveolar bone.

One of the limitations of the present study is that the resolution of the MDCT data collected from the study patients may be lower than that of CBCT. It is well known through many studies that neither MDCT nor CBCT measurements have any statistically significant difference from direct measurement and that both are highly accurate in assessing alveolar bone morphology [3, 13–17]. Loubele et al. measured the horizontal width of the mandibular premolar site in the dry alveolus and showed reliable values with both CBCT and MDCT, but showed that the MDCT values had about 0.5 mm greater error range than CBCT [18]. Fuhrmann et al. measured the amount of resorption of alveolar bone in dry skull using MDCT with a 1.0-mm slice thickness and found that it was highly accurate when compared with the actual measured values, with an overestimation of only 0.2 mm by MDCT [4].

## Conclusion

This preliminary study suggested the ideal alveolar bone morphology using three-dimensional imaging. Our results can be useful as a clinical reference when determining dental implant diameter and positioning. And these measurements may also be useful during the production of prefabricated 3D scaffold and customized alveolar bone restoration.

## Data Availability

Data sharing is not applicable to this article as no data sets were generated or analyzed during the current study.

## Abbreviations

CBCT: Cone beam computed tomography
CT: Computed tomography
DICOM: Digital Imaging and Communications in Medicine
MDCT: Multiple detector computed tomography
